# Some Nanocarrier’s Properties and Chemical Interaction Mechanisms with Flavones

**DOI:** 10.3390/molecules28062864

**Published:** 2023-03-22

**Authors:** Cecilia Espíndola

**Affiliations:** Department of Physical Chemistry, University of Seville, C/Profesor García González 1, 41012 Seville, Spain; carespedia@alum.us.es

**Keywords:** tropoflavin, baicalein, luteolin, organic NPs, covalent and non-covalent interaction, CNT flavonoids, nanomedicine, flavone chemistry and pharmacology, reaction mechanisms with functional groups

## Abstract

Flavones such as 7,8-dihydroxyflavone (tropoflavin), 5,6,7-trihydroxyflavone (baicalein), 3′,4′,5,6-tetrahydroxyflavone (luteolin), 3,3′,4′,5,5′,7-hexahydroxyflavone (myricetin)*,* 4′,5,7-trihydroxyflavone (apigenin), and 5,7-dihydroxyflavone (chrysin) are important both for their presence in natural products and for their pharmacological applications. However, due to their chemical characteristics and their metabolic processes, they have low solubility and low bioavailability. Knowledge about the physicochemical properties of nanocarriers and the possible mechanisms of covalent and non-covalent interaction between nanoparticles (NPs) and drugs is essential for the design of nanocarriers to improve the bioavailability of molecules with pharmacological potential, such as tropoflavin, baicalein, luteolin, myricetin, apigenin, and chrysin. The parameters of characterization of some NPs of these flavones, such as size, polydispersity index (PDI), zeta potential, encapsulation efficiency (EE), and % release/time, utilized in biomedical applications and the covalent and non-covalent interactions existing between the polymeric NPs and the drug were analyzed. Similarly, the presence of functional groups in the functionalized carbon nanotubes (CNTs), as well as the effect of pH on the % adsorption of flavonoids on functionalized multi-walled carbon nanotubes (MWCNT-COOH), were analyzed. Non-covalent interaction mechanisms between polymeric NPs and flavones, and covalent interaction mechanisms that could exist between the NPs and the amino and hydroxyl functional groups, are proposed.

## 1. Introduction

The application of advanced research in nanotechnology together with advances in biomedical and pharmacological sciences has allowed nanomedicine to evolve rapidly over recent decades. This has been achieved primarily through advances in nanocarrier design and research. According to their nature, nanocarriers are classified as organic, inorganic, and hybrid [1,2].

NPs based on proteins, polymers, liposomes, lipoplexes, and polyplexes, as well as macromolecular conjugates, nanoemulsions, and polymeric micelles, are organic in nature. Furthermore, inorganic nanocarriers include CNTs, quantum dots and metal, and metal oxide nanoparticles [3,4,5], among others.

Several mechanisms exist for the cellular internalization of nanocarriers, depending on their physicochemical properties. When the pharmaceutical target is located inside the cell, its pharmacological action takes place through mechanisms that involve its passage through the cell membrane in cell–cell or cell–specialized tissue interaction, for example, its passage through mucous membranes, epithelia, or endothelium; diffusing through the plasma membrane; or accessing the specific organelle where the biological target is located [6]. 

Nanocarriers’ functionality is influenced by their physicochemical properties, such as shape, size, loading surface, hydrophobicity, material, stiffness, and elasticity [7]. The effectiveness of nanocarriers has been demonstrated in the incorporation of therapeutic/diagnostic groups and markers with the control of their circulation inside the body and in the places of destination [8,9,10,11]. The internalization process also depends on the surface charge of the NPs in addition to the size. Electrostatic and van der Waals forces are important in interactions with biomolecules and cells. There is a correlation between the zeta potential and endocytosis/exocytosis mechanisms. 

Several factors characterize the biological environment of NPs, such as pH, ionic strength, oxygen level, organic matter, and the different components of the organic fluids surrounding the NPs once they enter the bloodstream. All these factors promote and define the type of interaction of the NPs with other NPs, with cells, with the surrounding environment, and with other nanomaterials, affecting in this way the PDI and zeta potential values.

The surface of NPs influences aggregation, stability, absorption [12], solubility [13], pharmacokinetics, distribution, colloid environment [14], plasma protein binding [15], blood–brain barrier crossing [16], elimination, accumulation, and cytotoxicity [17]. Therefore, any changes in the chemical, electrical, optical, magnetic, and surface properties of NPs have a significant influence on their functionality [18]. 

Functionalization is a chemical process by which functional groups are inserted into the walls of NPs [19]. Functional groups are generally utilized to anchor ligands to the surface of NPs. To increase stability, decrease toxicity, and modulate internalization mainly in biomedical applications, strategies such as surface modification of NPs with functional groups such as hydroxyl groups, carboxyl groups, and amine groups, through covalent interactions, in addition to the utilization of polyethylene glycol (PEGs), have been developed [17,20].

In NPs’ self-assembly, the most frequent non-covalent interactions are van der Waals forces and H-bonds. H-bonds are electrostatic dipole–dipole interactions between a hydrogen donor and acceptor with a directional and linear structure [21]. In addition to the above interactions, π-π stacking interactions and hydrophobic interactions are also present in the interactions between polymeric NPs and biomolecules. 

Structurally, CNTs are classified as single-walled (SWCNTs) and multi-walled (MWCNTs). Modification of CNT walls can be realized in a covalent manner when elements such as fluorine, oxygen, nitrogen, or biomolecules are directly incorporated into the CNT walls. Non-covalent modification can occur by strategies that include the adsorption of surfactants, biological macromolecules, and polymers, without involving modification of the CNT structure [22]. 

After covalent functionalization, the electrical, optical, and mechanical properties of CNTs change. These changes improve the solubility and dispersion of CNTs in different polymers and solvents. The functionalization realized with polymers or surfactants preserves the integrity and physical properties of CNTs, improving their solubility and processability. However, the main disadvantage of this type of functionalization is that the forces between the surrounding molecule and the CNTs are weak, and the charge transfer efficiency could be low [23]. In biomedical applications, CNTs are generally covalently or non-covalently functionalized with PEGs or other biomolecules [22].

Several techniques are utilized to identify CNT surface changes after the functionalization process: transmission electron microscopy (TEM) [24,25]; infrared spectroscopy with Fourier transform (FT-IR) [26,27,28], HPLC [29,30], X-ray photoelectron spectroscopy (XPS), [25,31], differential scanning calorimetry (DSC) [25], field emission scanning electron microscopy (FESEM) [32], and energy dispersive X-ray (EDX) [21].

Flavones are a group of flavonoids possessing the B-ring connected at the C2 position, a double bond between C2 and C3, and a carbonyl group at C4. This structure gives the molecule certain chemical characteristics that confer its own biological and pharmacokinetic properties. 

**Figure 1:**
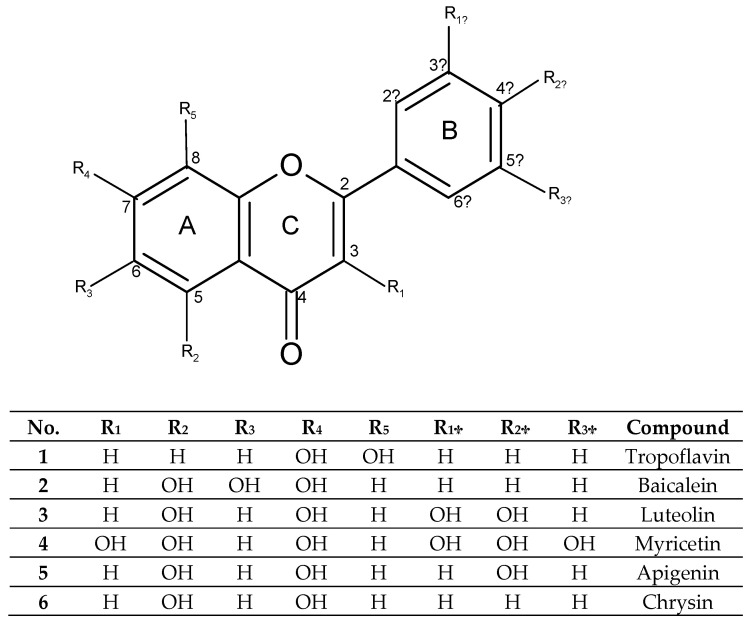
Structure selected flavones.

Flavones such as tropoflavin, baicalein, luteolin, myricetin, apigenin, and chrysin (Figure 1) are studied mainly for their properties as pharmacological agents. 7,8-Dihydroxyflavone (tropoflavin **1**) is a flavone found in species such as *Godmania aesculifolia*, *Tridax procumbens*, *Primula farinosa* L., and *Chrysanthemum morifolium* [33]. As a neuroprotective agent, the presence of 7,8-OH groups in its structure makes it a chemical agent that emulates the biochemical and physiological action of brain-derived neutrophic factor (BDNF) and serves as a selective agonist of the tropomyosin-related kinase B receptor (TrkB) [34,35]. The anticancer activity of tropoflavin has been demonstrated by Liu et al. (2020) [33], as an inhibitor of human ornithine decarboxylase (ODC), in in vitro studies. Similarly, it has been shown that tropoflavin had anti-enterovirus (EV71) activity at a concentration of 50 Μm. It inhibits 40% of viral IRES-internal ribosome entry site (IRES) activity by interfering with virus replication [36,37]. 

The presence of tropoflavin in plasma is detectable after 8 h (5 ng/mL) of administration. In vivo metabolism study shows that tropoflavin undergoes glucuronidation, sulfation, and methylation. Among these modifications, glucuronidation and sulfation are mainly responsible for the in vivo elimination of flavonoids [34] and their low bioavailability. 

Baicalein **2**, a well-known bioactive natural flavone, has been identified in several plant species including *Scutellaria baicalensis* Georgi; *Scutellaria barbata* D. Don; *Scutellaria lateriflora* L.; *Oroxylum indicum* (L.) Kurz, and the fungus *Tuber aestivum* Vittad [38,39]. 

Baicalein has been demonstrated to have antitumor [40,41,42,43], antiviral [44,45], antimicrobial [46], anti-inflammatory [47], antioxidant [48,49,50], neuroprotective, and anti-IR-insulin resistance activity [51,52]. In addition, it is hepatoprotective and has an effect both on cardiovascular and cerebrovascular systems and on bone [53], and gastrointestinal disease [54]. 

Baicalein at a dose of 20 mg/kg, 5 d/week for 21 days can inhibit MDA468 breast cancer by 40%, which is comparable to the positive effect of the drug cisplatin (5 mg/kg) [55]. Baicalein has also shown anticancer effects on hepatocellular carcinoma. It does this by modulating associated molecules and signaling pathways resulting in inhibition of cell proliferation, induction of apoptosis, cell cycle arrest, and induction of autophagy [42]. Xiaoling et al. (2018) [56] evaluated the effect of baicalein on cervical cancer cells and other cancer types, they concluded that baicalein arrests the cell cycle in the G_0_/G_1_ phase and cyclin D1 decrease through the AKT-GSK3β signaling pathway [43]. Baicalein was able to reduce endometriosis by suppressing the viability of human endometrial stroma in cells in vitro [57]. 

Similarly, baicalein showed antioxidant activities against the hydroxyl radical, the 2,2-diphenyl-1-picrylhydrazyl (DPPH) radical, and the alkyl radical, with IC_50_ values of 10–32 IM [48]. Baicalein (50 μM) also exhibited antioxidant activity in the cardiomyocyte ischemia/reperfusion model, decreasing subsequent cell death from 52.3 to 29.4% [41].

In studies carried out by Chen et al. (2017) [53], it was found that baicalein affects the osteogenic differentiation of human periodontal ligament cells (hPDLCs). These cells are important in periodontal tissue regeneration, decreasing the growth of hPDLCs, increasing alkaline phosphatase and calcium deposition in a dose-dependent manner.

Błach-Olszewska et al. (2008) [58], demonstrated that baicalein regulates natural innate immunity by modulating cytokine production and stimulating resistance in human leukocytes. Sithisarn et al. (2013) [47] evaluated the effect of baicalein on human lung epithelial cells (A549) infected with avian influenza strain A/Thailand/Kan-1/04 and found that baicalein inhibited the production of virus proteins and the viral replication cycle. Similarly, baicalein has been shown to have antiviral effects against replication of the mosquito-borne Chikungunya virus (CHIKV), which causes disabling arthritis in infected individuals. It is considered that baicalein exerts its antiviral action in the first hours of treatment and in the early stages of infection, decreasing the production of CHIKV proteins at concentrations of 100 µg/mL [45]. 

In addition to the above pharmacological applications, baicalein also a strong synergistic effect with penicillin G/amoxicillin against 20 penicillin-producing clinical strains of *S. aureus* [46]. 

Baicalein has a low bioavailability due to its poor water solubility, being a class IV compound according to the Biopharmaceuticals Classification System (BCS) (solubility: 0.052 mg/Ml; lipophilicity: Papp = 0.037 × 10^−6^ cm/s) [59]. The low availability of baicalein may be due to the transformations it undergoes following the processes of glucuronidation and sulfation through the small intestine. For this reason, baicalein is considered a drug with a presystemic or first-pass metabolism, since when the drug is administered orally, the active ingredient of the drug is considerably reduced before it reaches the circulatory system [60].

3′,4′,5,7-Tetrahydroxyflavone (luteolin, **3**) is a flavone found in numerous species of the plant kingdom. It is found in botanical families including the phyla Bryophyta, Pteridophyta, Pinophyta, and Magnoliophyta [61]. Luteolin has also been found in edible plants such as carrot (*Daucus carota*), capsicum (*Capsicum annuum*), celery (*Apium graveolens*), olive oil (*Olea europaea*), rosemary (*Rosmarinus officinalis*), mint (*Menta x piperita*), thyme (*Thymus vulgaris*), oregano (*Origanum vulgare*), lettuce (*Lactuca sativa*), chocolate (*Theobroma cacao*), and cucumber (*Cucumis sativus*) [62], among others. Luteolin is present in plants as an aglycone (Figure 1) and as glycosides. 

Epidemiological studies have found that the consumption of high levels of luteolin decreases the risk of developing some chronic diseases [61]. Several studies have demonstrated luteolin’s activities as an antioxidant [63], anti-inflammatory [64], anti-cancer [65], and chemotherapy agent [66]. Castellino et al. (2019) [67] evaluated the effect of luteolin supplementation on cardiometabolic risk factors in human patients and found an improvement in vascular function through dilation of the brachial artery. Luteolin also has other biological activities [68]. 

Myricetin **4** is a hexahydroxyflavone with hydroxyl groups at positions 3,3′,4′,5,5′, and 7-OH. It is found in plant species such as *Myrica rubra*, *Ficus auriculata*, *Visnea mocanera*, *Rosa canina* L, *Portulaca oleracea* L., *Urtica dioica* L., and other plants and organisms such as *Saccharomyces cerevisiae* [69]. 

The pharmacological activities of myricetin have been reviewed by [70] and [71]. Myricetin exhibits anticancer [72], antimicrobial [73], antioxidant [74], and antidiabetic [75] effects, among others. 

Apigenin **5** is 4′,5,7-trihydroxyflavone. It is a flavone widely distributed in plants of the Asteraceae family, mainly *Matricaria* [76], *Achillea* [77], and *Artemisia* [78] genera or in some genera of the family Lamiaceae, such as *Sideritis* [79]. Apigenin is a flavone known mainly for its importance in the prevention of various types of cancer [80,81,82]. It also has antioxidant [83], anti-inflammatory [84], and antiviral [85] properties.

Chrysin **6** is a 5,7-dihydroxyflavone, which is found in honey, propolis, and in some flowers and fruits of species such as *Juglans regia* [86], *Hyphaene thebaica* [87], and *Cytisus villosus* [88], having several effects on human health [89]. However, chrysin also exhibits low bioavailability due to its rapid metabolism in the gastrointestinal tract, as observed in in vivo studies after oral administration of 400 mg chrysin in humans [90]. 

Tropoflavin, baicalein, luteolin, myricetin, apigenin, and chrysin are flavones that have low bioavailability due to their low solubility in water. The enhancement of the drug-like properties of natural compounds, such as their bioavailability, targeting, and controlled release, has been achieved through the incorporation of nanoparticles. However, increasing the bioavailability of a drug also depends on the chemical interactions that occur between the functional groups of the chemical compound itself with cell surface molecules either through cell–cell, cell–tissue, and cell–surrounding environment interactions. Properties of some NPs of these flavones, the covalent and non-covalent interactions mechanism between the NPs and drugs, as well as the effect of factors such as pH on the functionalized MWCNT-COOH, have been reviewed and analyzed. Some mechanisms of non-covalent and covalent interaction with the functional groups have also been proposed. 

## 2. NPs—Flavones

### 2.1. 7,8-Dihydroxyflavone

Chen et al. (2020a) [91] encapsulated 7,8-dihydroxyflavone (tropoflavin) in liposomes with phosphatidylcholine and glycerol at ratios of 1:40, 1:30, 1:15, 1:10, and 1:5. For the concentration ranges 1:40–1:5, the size of the tropoflavin liposomes was 107.50–247.27 nm, and the PDI and zeta potential were 0.126–0.379 and −19.57–−22.67 mV, respectively. The EE was 89.50%. Regarding the effect of pH on the stability of tropoflavin liposomes, it was observed that at pH values of 2–12, the size of liposomes presented relative stability, due to the protection provided by the structure of phospholipids and cholesterol to the liposomal membrane from the effects of acidic pH. At a tropoflavin concentration range of 1–100 μM, the tropoflavin liposomes do not exhibit cytotoxicity and were used in transmembrane transport assays. However, the same authors in another work [92] obtained an EE of 98.31% for tropoflavin-zein-lactoferrin NPs, with a NP size of 82.3 ± 1.01 nm. On the other hand, Prasanna et al. (2021) [93] encapsulated tropoflavin in gold NPs, which presented a size of 35 nm and a zeta potential of −34.1 mV. 

**Figure 2:**
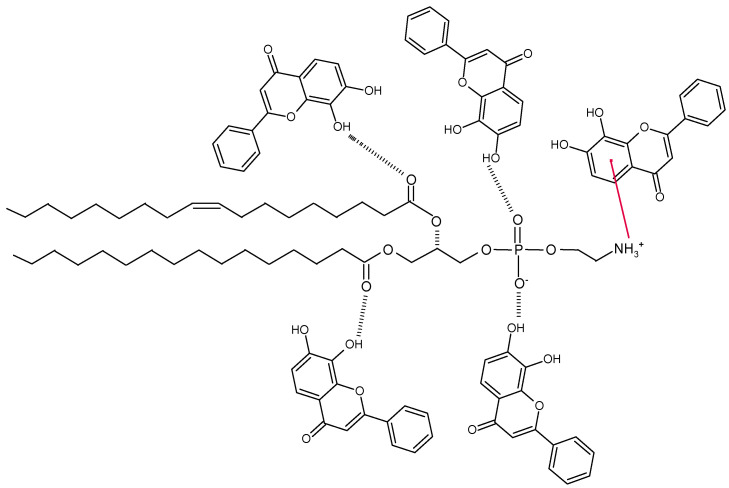
Non-covalent interaction mechanism between tropoflavin and phosphatidylcholine in liposome formation.

Since flavones are non-polar, they should be confined to the hydrocarbon region of the lipid membrane; however, in a study by Scheidt et al. (2004) [94], chrysin, luteolin, and myricetin molecules presented a rapid reorientation in the membrane, detecting a maximum distribution at the lipid/water interface. Thus, the hydroxyl groups of the flavones participate in the bond networks of the lipid–water interface. Therefore, the interaction between flavone and lipid occurs via the H-bond between the OH groups of the flavone with both the C=O groups of the lipid and the phosphate group. A π–cation interaction occurs between the NH_3_^+^ group of the phospholipid and the A-ring of the flavone (Figure 2). In addition to the above, other favorable interactions between the flavone and the lipid molecule involve charge–dipole and dipole–dipole interactions. 

### 2.2. 5,6,7-Trihydroxyflavone

With respect to the characterization of the 5,6,7-trihydroxyflavone (baicalein) NPs, it has been found that the size of mesoporous silica NPs (MSNs) is considerably larger, 367 ± 94 nm [95], than the size of liposomes 135–154 nm [96] or NPs 82.5 ± 1.7 nm [97]. Regarding the variation of the size of NPs in the NP population that is PDI, it is observed that nanoemulsions (NE) and NPs are monodisperse, while liposomes vary in size. The surface charge of baicalein encapsulated in NE and represented by the zeta potential is very low −22.4 ± 3.1 mV [98], indicating nanoaggregates formation, as in mesoporous silica NPs (MSNs), in contrast to liposomes and NPs, where high potential values, such as −1.89 – −2.11 mV and −1.5 ± 0.4 mV respectively, indicate suspension stability. Baicalein liposomes’ stability was homogeneous for more than two weeks, and its in vivo stability was affected by the interaction with other proteins and between lipoproteins present in the circulation. Encapsulated baicalein increased the cell viability of Hs68 fibroblasts by reducing their cytotoxicity. Regarding EE, a decrease from 33.65 to 25.40% occurred when baicalein concentration was increased from 30 to 80 μg/mL and was much lower than the EE that occurred for NE, 98%, and NPs, 86.2% (Table 1).

Baicalein liposomes containing mainly phosphatidylcholine have a neutral surface, and the zeta potential was around 0. The presence of baicalein does not alter the electrophoretic mobility of the liposomes. Fang et al. (2018) [96] concluded that the reduction in particle size may be mainly due to strong interactions with baicalein via the H-bond but has no effect on the zeta potential. 

Baicalein has low solubility in aqueous solution and low bioavailability in vivo, which limits its application; liposomal nanoencapsulation is recommended due to its high compatibility and easy incorporation efficiency [96]. Therefore, drugs encapsulated in NPs present a reduced dispersion of the drug, longer permanence time on the skin, and facilitate the internalization of the drug into the cells [99]. Similarly, because of their ease of preparation, handling, biocompatibility, and biodegradability, NEs are a model for drug delivery [100]. 

In cancer chemotherapy and other diseases, several strategies have been developed to improve the transport, delivery, solubility, and bioavailability of drugs from bioactive or synthetic compounds. To improve these properties, mainly solubility and bioavailability, conventional nanocarriers such as liposomes, micelles, nanocrystals, and polymeric NPs have been designed. In the case of baicalein, several nanocarriers have been developed to improve the low solubility and bioavailability (Table 2).

**Figure 3:**
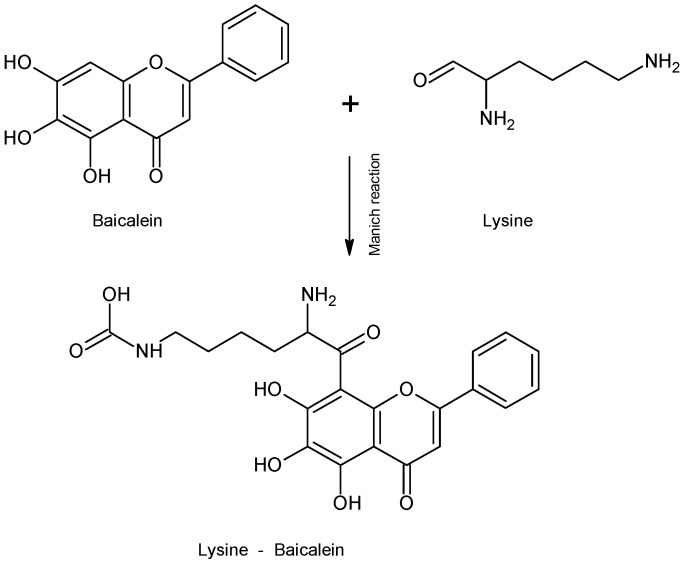
Reaction of Lysine—Baicalein complex formation.

It is important to highlight the studies carried out on the development of delivery systems based on conjugated nano drugs or self-assembled NPs. In a study carried out by Wang et al. (2015) [97], they developed self-assembled hyaluronic acid-lysine-baicalein (HA-L-BCL) NPs and evaluated their anticancer effect on A549 human lung cancer cells. Firstly, the L-BCL complex was synthesized by the Mannich reaction (Figure 3). Subsequently, the N-hydroxysuccinimide-lysine-baicalein (NHS-L-BCL) system was synthesized in the presence of 1,4-diamino butane as reactant and sodium cyanoborohydride (NaCNBH_3_) as reagent and then HA-L-BCL NPs were prepared in the presence of poly-(lactic-*co*-glycolic acid) (PLGA).

**Figure 4:**
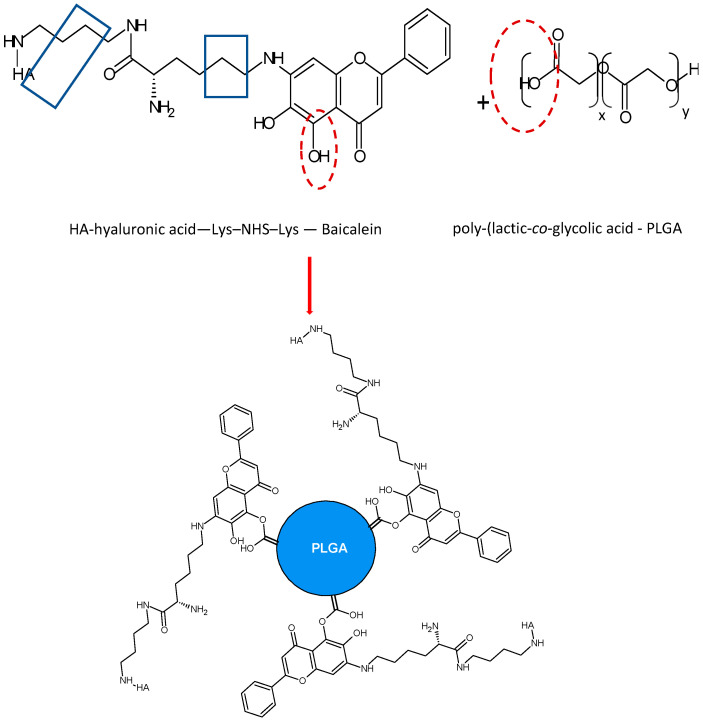
Non-covalent interaction mechanism in HA-L-BCL-PLGA complex formation.

The non-covalent interaction for HA-L-BCL-PLGA complex formation can be presented as the following: HA-Lysine interaction is H-bond type interaction between the H―C=O carbonyl group of HA and the NH_2_ group of lysine (Figure 4).

The interaction between baicalein and lysine could be by the H-bond type between the NH_2_ group of lysine and the B-ring 7-OH group of baicalein or between the C=O group of lysine and the same 7-OH group of baicalein. An H-bond interaction can also occur between the alkylammonium ion (-CH_2_-NH_3_^+^) of lysine and the 6-OH group of baicalein as in the scheme above.

In the HA-L-BCL-PLGA polymeric complex, an H-bond interaction can occur between the carbonyl group of the C-ring of baicalein and the carboxyl group of PLGA or between the 5-OH and 6-OH groups of baicalein and PLGA as depicted Figure 4.

According to Pool et al. (2012) [101], a negative ζ potential value at pH 7.0 of the polymeric NPs of some flavonoids may be due to the presence of the ionized carboxyl groups of the PLGA matrix. In the same way, the presence of each specific flavonoid changes the charge of the polymeric NPs, without altering the stability of the NPs. Values of ζ between ≈+30 and ≈−30 mV exert enough electrostatic repulsive force to prevent aggregation of the nanoparticles. The interactions that exist between the flavonoid and the PLGA matrix are non-covalent such that each flavonoid is dispersed in a non-crystalline state within the polymeric PLGA matrix.

The utilization of polymers with PLGA has advantages such as chemical and mechanical stability, ease of modification and adjustment of properties, as well as low non-specific protein binding. However, it has disadvantages such as the possibility of polymer toxicity and non-biodegradability [102].

HA-L-BCL NPs were prepared by nanoprecipitation. BCL NPs were characterized and the EE for BCL NPs was 86.2 ± 2.7% (Table 1). Since the bonds formed with amino acids are weak, the use of amino acids such as lysine as ligands is recommended in the production of prodrugs as it facilitates drug release. Similarly, although PEG is a widely used connector for drug development, its efficiency has been demonstrated to be lower in relation to the individual drug. In HA-L-BCL NPs, its amphiphilic character allows it to self-assemble in such a way that the hydrophobic center is BCL and the hydrophilic shell is HA. At 50–200 µM concentration (*p* < 0.05), the cytotoxic effect of HA-L-BCL NPs was higher than that of the other formulations tested and at this same concentration, the IC_50_ of the BCL NPs was 0.517 [97]. 

### 2.3. 3′,4′,5,7-Tetrahydroxyflavone

Dang et al. (2014) [103] encapsulated 3′,4′,5,7-tetrahydroxyflavone (luteolin) in solid lipid nanoparticles (SLNs). Liu et al. (2022) [104] obtained EE values of 74.80 and 76.4% when encapsulating luteolin in methoxy poly(ethylene glycol)–poly(lactic-co-glycolic acid) (mPEG-PLGA)-luteolin coated with a pH-dependent copolymer Eudragit S100 (Table 3). Although the percentage of EE is very similar, the size difference of 47 ± 0.51 and 197.45 ± 20.09, respectively, is notable, in addition to the difference in zeta potential of −9.62 and −23.5 ± 1.16. Ding et al. (2020) [30] encapsulated luteolin in poly(lactic-co-glycolic acid) PLGA NPs and modified them with Her-2 antibody to produce immunolabeled microspheres. They worked with SGC-7901 gastric cancer cells. The size and ES for PLGA-luteolin were 184 nm and 91.80 ± 6.2% and for Her-2-PLGA-luteolin, 203 and 90.4 ± 6.1%, respectively. The modification with the antibody altered the surface of PLGA-luteolin. However, although PLGA-luteolin has strong inhibitory activity on cancer cell proliferation, this effect was enhanced by Her-2-PLG-luteolin.

A similar EE of 90.2% was obtained by Puhl et al. (2011) [105] by elaborating luteolin nanocapsules and nanospheres with the polymers: polymer [poly(-caprolactone) (PCL) and poly(lactic-co-glycolic acid)] (PLGA) with the natural oil isodecyl oleate.

A high EE (98.32%) was reported by Qiu et al. (2013) [106] for luteolin micelles encapsulated in the copolymer monomethoxy poly(ethylene glycol)-poly(ε-caprolactone) (MPEG-PCL)-luteolin. Poly(ε-caprolactone)/poly(ethylene glycol) (PCL/PEG) can self-assemble into NPs with a hydrophobic PLC center and hydrophilic PEG outside. In such a way, when a hydrophobic drug is encapsulated, the PLC and the drug will constitute the hydrophobic center, while the outside will be constituted by the hydrophilic PEG, thus originating an injectable intravenous drug. However, an EE of 51.6% was obtained by Qing et al. (2017) [107] when they functionalized luteolin micelles with various copolymers (methoxy polyethylene glycol-poly(lactic-co-glycolic acid) (mPEG_5K_-PLGA_10K_)-luteolin).

Tawornchat et al. (2021) [108] obtained an EE of 89.3% in luteolin NPs by enzymatic polymerization using H_2_O_2_ as the reagent, polyphenol oxidase (PPO) as catalyst, and PEG as the matrix. They found that the presence of the oxidative enzyme—horseradish peroxidase (HRP) was necessary for the chemical transformation. Although the anti-inflammatory activity of luteolin NPs is dose-dependent, there is no cytotoxicity at high doses unlike the cytotoxicity exhibited by the drug luteolin.

**Figure 5:**
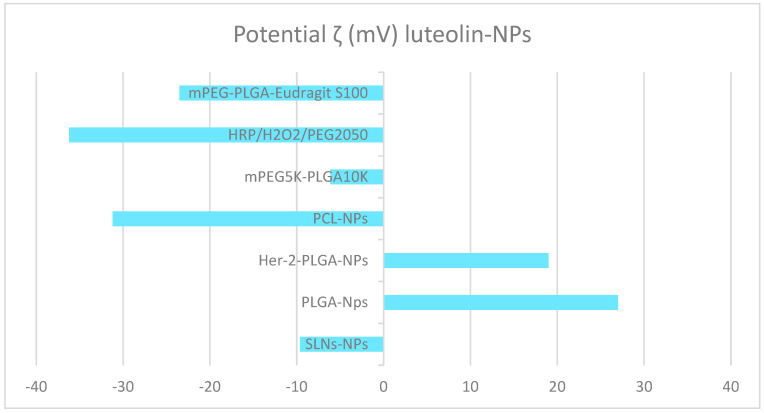
Zeta potential of some luteolin polymeric NPs.

Regarding zeta potential, both luteolin PCL-NPs [30] and luteolin-HRP/H_2_O_2_/PEG2050 [108] present similarly low values (−31.2 ± 0.7 and −36.2 ± 0.2), respectively. In contrast to the high zeta potential obtained for mPEG_5K_-PLGA_10K_ luteolin (−6.12) [107], and −9.62 obtained for solid lipid nanoparticles (luteolin SLNs) [103]. Studies by Ding et al. (2020) [30] obtained zeta potential values of 27 and 19 for PLGA-luteolin NPs and Her-2-PLGA-luteolin NPs, respectively (see Figure 5). 

PLGA-luteolin liposomes presented a size of 99.1 ± 5.75 nm [109] in contrast to PLGA-luteolin NPs [30] whose size was 184 nm, unlike tropoflavin and baicalein liposomes, whose sizes were 107.50–247.27 nm and 135–154 nm, respectively.

Table 4 compares the size, PDI, zeta potential, and EE of tropoflavin, baicalein, and luteolin liposomes.

PDI values of tropoflavin 0.126–0.379 and luteolin 0.198 indicate that the liposome sample is monodisperse, while the baicalein PDI 0.462–0.503 liposomes exhibit a variety of sizes. The ζ potential values of −19.57– −2267 mV for tropoflavin and −12.5 mV for luteolin indicate the liposome sample presents floccules or aggregates while a value of −1.89–−2.11 mV for baicalein indicates stability in the suspension. Regarding the EE values for the three flavones presented in Table 4, values of 89.50 and 85.6% are presented for tropoflavin liposomes and PLGA-luteolin liposomes, respectively, while an EE of 25.40–33.65% is presented for baicalein liposomes.

The drug release depends on the size of the NPs, the trapping efficiency, the composition, and the biodegradation of the NPs [110]. 

**Figure 6:**
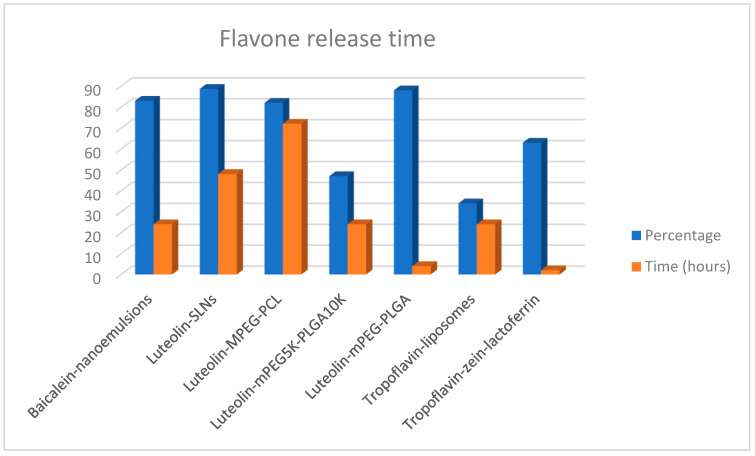
Release of tropoflavin, baicalein, and luteolin from NPs at pH 7.4 and 37 °C.

When the relationship between the type of NPs and the release time of these flavones was analyzed (Figure 6) it was found that for the flavones tropoflavin, baicalein, and luteolin, the release time of the NPs was 24 h to pH 7.4 at 37 °C. However, it is important to highlight that the release percentage of baicalein from nanoemulsions was 83% [98] with respect to the 47% release of luteolin from mPEG_5K_-PLGA_10K_ [107] and the 34% release of tropoflavin from liposomes [91]. 

The highest release time of 72 h was presented by luteolin release from MPEG-PCL with 82.5% [106], while tropoflavin was released at 2 h from zein-lactoferrin NPs with 63% [92]. The same percentage of 88% release of both SLNs [103] and mPEG-PLGA [104] was presented at 48 and 4 h, respectively. NPs’ pegylation with PEG provides a convenient method to provide high colloidal stability to NPs in physiological media and to avoid undesirable interactions of NPs with proteins or other blood components [111]. Luteolin release from MPEG-PCL was 49.3% at 12 h and 82.5% after 72 h [106] at pH 5.7 and at 35 °C, and 89.1% luteolin release from PCL NPs was obtained [105].

### 2.4. 3,3′,4′,5,5′,7-Hexahydroxyflavone

Sims et al. (2020) [112] evaluated the cationic interactions between myricetin, a flavone with hydroxyl groups at the 3, 3′, 4′, 5, 5′, 7 positions, and polymeric nanoparticle carriers (NPCs). They found that flavone–NPC interaction was influenced by H-bond, π-π interactions, and van der Waals forces. Electrostatic forces between the tertiary amines in the NPC corona and the myricetin-specific hydroxyl groups stimulate π-π interactions between the unsaturated rings and enhance the conjugation between the aromatic compounds enabling π-π* transitions.

### 2.5. 4′,5,7-Trihydroxyflavone

Zhai et al. (2013) [113] elaborated apigenin-polymeric micelles by thin-film dispersion method to evaluate their effect on human liver hepatocellular carcinoma (HepG2) cells and human breast cancer cell line MCF-7 (Table 5).

H-bonding can occur between the hydroxyl groups of apigenin and the carboxyl of the PEG chains in the apigenin-conducting micelles, leading to a decrease in micelle size compared to the non-apigenin micelles which was 18.9 nm. Such interactions are also responsible for the negative value of the zeta potential of the micelles. Apigenin release from the polymeric micelles was about 85% at 50 h and at 37 °C. They concluded that the polymeric micelles have a greater cytotoxic effect on MCF-7 cells than on HepG2 cells.

Ganguly et al. (2021) [114] elaborated apigenin NPs such as apigenin-poly(Lactic-co-glycolic acid) nanoparticles (API-PLGA-NPs) and apigenin-galactose-nanoparticles (API-GAL NPs) and evaluated their effect on human liver hepatocellular carcinoma (HepG2) cells. Their internalization, apoptotic, and cytotoxic potential of both free apigenin and apigenin NPs were measured. The release of apigenin from API-NPs was 88% and from API-GAL-NPs was 86% after 8 days at pH 7.4. NPs’ stability was confirmed for 90 days at 4 °C. They concluded that galactosylation of apigenin NPs significantly enhances the internalization of apigenin NPs into HepG2 cells, possibly due to the presence of asialoglycoprotein receptors on the surface of HepG2 cells, thus improving the apoptotic and cytotoxic effects of API-GAL-NPs on both API-NPs and API-PLGA-NPs.

### 2.6. 5,7-Dihydroxyflavone

Siddhardha et al. (2020) [115] elaborated chrysin chitosan nanoparticles (CCNPs) to evaluate their anti-biofilm effect against *Staphylococcus aureus*. The size of CCNPs was 130–341 nm, PDI 0.487, EE 80.86%, and percentage release was 90.5% at 10 h, pH 7.4, and at 37 °C. They found that CCNPs inhibited the early stages of biofilm development. For their part, Ragab et al. (2022) [116], evaluated the effect of chrysin chitosan CCNPs nanoparticles on the activity of the enzyme succinate: ubiquinone oxidoreductase of mitochondrial complex II in human fibroblasts. The size of CCNPs was 49.7 ± 3.02 nm, with a positive zeta potential between +35.5 and +77.02 mV, an EE of 92.63%, and a release percentage of 90% at 18 h, pH 7.4, and at 37 °C. They found that the inhibitory effect on the activity of the enzyme succinate: ubiquinone oxidoreductase of mitochondrial complex II was greater with chrysin than with CCNPs in human fibroblasts. 

### 2.7. Other Flavonoids

**Figure 7:**
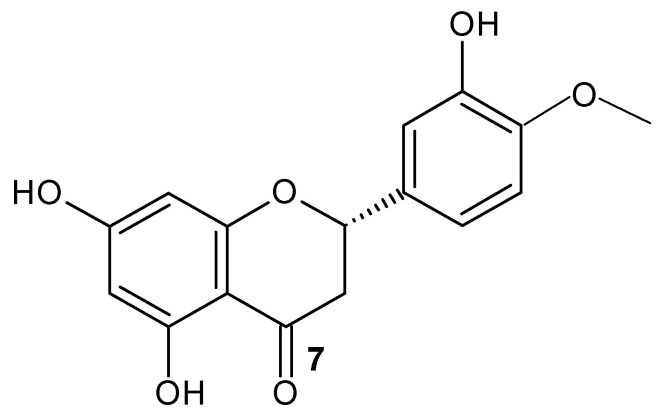
Hesperetin structure.

Aiello et al. (2019) [117] evaluated the utilization of flavonoid NPs for cancer therapy. Further studies with flavonoid nanoparticles have been carried out; for example, Ersoz et al. (2019) [118] evaluated the anticancer activity of the flavanone hesperetin (**7**) (Hsp) (Figure 7) and hesperetin nanoparticles (HspNPs) coated by PLGA polymer on C6 glioma cells. The HspNPs’ size was 260 ± 20 nm and PDI was 0.355 ± 0.006; this value indicates that the sample was monodisperse. Regarding the ζ, the potential value was −42 ± 1.3 mV, and this highly negative value indicates that floccules or aggregates were formed, taking the colloidal form. They identified that HpsNPs present anti-proliferative and apoptotic activity on C6 glioma cells.

The size difference of the PLGA-NPs of hesperetin 260 nm, with respect to luteolin 184 and 110 nm of apigenin, is noteworthy. The same as the PDI value of apigenin 0.041 ± 0.004 with respect to the PDI of hesperetin 0.355 ± 0.006, indicating the high homogeneity of the size of the HpsNPs. The apigenin and hesperetin PLGA-NPs suspension formed floccules or aggregates, unlike the luteolin PLGA NP suspension, as indicated by the zeta potential values reported in Table 6. 

With respect to the utilization of inorganic NPs, Kollur et al. (2021) [119] prepared zinc oxide NPs functionalized with luteolin and evaluated their anticancer activity in human MCF-7 breast cancer cells. The size of Lu-ZnONPs was 17 nm. In the formation of Lu-Zn ONPs, an interaction occurred between the 3′,4′-OH groups of the luteolin B-ring, which are oxidized by the reduction of Zn ions to ZnONPs, due its ability to donate electrons. At a concentration of 40 μM of Lu-ZnONPs, only 15% of MCF-7 cells remained viable. 

AgNPs have been synthesized by different chemical, photochemical, electrochemical, and biological methods, as well as through organic synthesis or green synthesis of nanoparticles [120]. Qing et al. (2017) [121] prepared AgNPs using methoxy polyethylene glycol mPEG luteolin with a reducing agent and stabilizer without additives. They evaluated the antibacterial action of the mPEG-luteolin-AgNPs complex on *Staphylococcus aureus*, *-Lactamases Staphylococcus aureus*, *Escherichia coli,* and *y-Lactamases Escherichia coli*. Furthermore, they evaluated the cytotoxicity of the mPEG-luteolin-AgNPs complex on human neuroblastoma SK-N-SH and normal HVEC cells. The mPEG-luteolin-AgNPs presented a size of 25 nm and a zeta potential of −25.5 mV. The Ag^+^ ions were reduced to metallic Ag^0^ by the adjacent hydroxyl of the mPEG-luteolin, which is a reducing agent. So, the multiple Ag atoms collide with each other and form a crystalline Ag core that adsorbs the Ag ions and forms a colloidal [Ag]_m_nAg^+^ core. Furthermore, mPEG-luteolin is strongly adsorbed on the surface of AgNPs, thus reducing their surface activity, preventing flocculation, and remaining monodisperse due to the presence of mPEG-luteolin as a stabilizing agent. They concluded that mPEG-luteolin-AgNPs exhibit a better inhibitory effect on large negative bacteria at low concentrations and exhibit toxicity on human neuroblastoma SK-N-SH cells in a dose-dependent action.

Organic synthesis of AgNPs has been performed from extracts of numerous plants that have been utilized as reducing and stabilizing agents for nanoparticles. Kobylinska et al. (2020) [120] developed a methodology to elaborate green synthesis of AgNPs from extracts of adventitious roots of *Artemisia* spp. and evaluated their antimicrobial activity. They concluded that the solvent, composition, and concentration of reducing agents present in the plant extracts directly influence the characterization parameters of the NPs and their antimicrobial activity.

**Figure 8:**
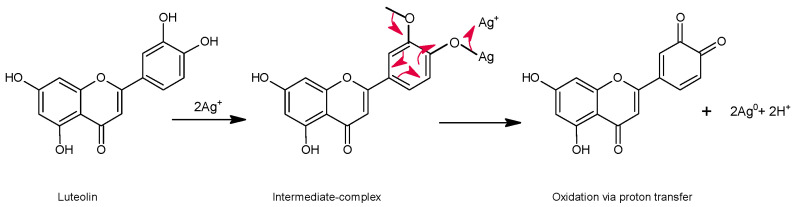
Proton transfer reaction mechanism in the flavone NPs.

In the interaction of AgNPs with flavonoids, the presence of hydroxyl groups with high reducing activity can reduce Ag^+^ ions through the formation of an intermediate complex followed by oxidation through the abstraction of an H atom (see Figure 8). In this process, two states of the formation of AgNPs have been recognized: the nucleation state and the growth state that finally gives rise to AgNPs. Similarly, in the sequence of reactions that initiate the Ag^+^ ions, the formation of radical oxygen species -ROS is involved, which, when reacting with antioxidants such as flavonoids, originate oxidation or decomposition compounds. On the other hand, the presence of biomolecules allows the formation of a protective cover over the AgNPs that acts as a stabilizing agent for the nanoparticles [120]. 

Bi et al. (2021) [122] encapsulated luteolin at various concentrations of 34, 68, and 102 mg in emulsions to make chitosan thin films. Nanoparticles containing luteolin 0.34%, 0.68%, and 1.02% (w/v) presented very similar sizes: 188.2 ± 1.1, 196.4 ± 1.6, and 198.2 ± 1.3 nm, respectively, indicating that the size of the NPs increases with increasing luteolin concentration. Regarding the PDI values obtained of 0.277, 0.213, and 0.258, respectively, these mean that the luteolin concentration of 68 mg may influence the homogeneity of the nanoemulsions. The zeta potential values −33.3 ± 1.5, −39.8 ± 2.3, and −0.3 ± 0.1 mV were negative because of the anionic character of glycerol monooleate. The zeta potential of −39.8 ± 2.3 mV indicates that with 68 mg of luteolin a high surface charge is obtained. Regarding the EE for luteolin concentrations of 34, 68, and 102 mg, values of EE 79.98, 89.52, and 74.15% were obtained, respectively, indicating that the maximum electrostatic repulsion was obtained with 68 mg of luteolin, which avoided the agglomeration of the nanoemulsion at this concentration. 

## 3. CNTs

The functionalization processes of CNTs generally involve the breaking of C=C bonds by oxidation. Among the techniques utilized for oxidation are wet chemistry, photooxidation, oxygen plasma, and gaseous treatment methods. The wet chemistry technique consists of the functionalization of CNTs with carboxyl -COOH and hydroxyl -OH groups [32]. 

Table 7 reports the band shifts corresponding to the -OH, C=O, C=C, and C=O functional groups obtained from FT-IR studies performed on functionalized MWCNT-COOH.

In the functionalization of MWCNTs with HNO_3_/H2SO_4_ acids, agglomeration was observed after 30 min of functionalization. This agglomeration was attributed to the hydrophobicity of the graphene sidewalls and π-π interactions between the individual CNTs [32]. However, the dispersed solution was formed due to the positively and negatively charged functional groups on the walls of the CNTs, which causes repulsion between them [19]. The treatment with excess acid causes the cleavage of C=C bonds in graphene CNTs, generating functional groups at the open ends of nanotubes and cut nanotubes. Similar results were observed by Yudanti et al. (2011) [124] when functionalizing MWCNTs with HNO_3_/H2SO_4_ acids. Possibly such an effect is caused by the acidic environment that produces a higher number of oxidation sites on the carbon atom by the exfoliation of graphite [125]. In addition, the increase of -COOH groups on MWCNTs caused by prolonged sonication leads to the disintegration of CNTs, making them shorter and thinner and converting them into amorphous carbon. This is possibly due to a disruption of the π electronic system of the CNT, which originates a degradation of the charge mobility and its mechanical properties [32].

Osorio et al. (2008) [19] evaluated several acid functionalization procedures with HNO_3_/H_2_SO_4_ and HCl on SWCNTs and MWCNTs. In the FT-IR studies, they found a band at 1600 cm^−1^ corresponding to C=C, and a band between 2800 and 3500 cm^−1^ that corresponds to C-H and O-H related to carboxyl and hydroxyl groups. The band at 1450 cm^−1^ corresponding to C-O indicates the presence of carboxyl groups on the oxidation surface and the band at 620 cm^−1^ indicates the presence of the H-bond. In turn, bands at 1640 cm^−1^ corresponding to the C=O group and 1560 cm^−1^ corresponding to C-O-C- were found by Park et al. (2015) [27] when they functionalized MWCNT with HNO_3_/H_2_SO_4_ acids.

**Figure 9:**
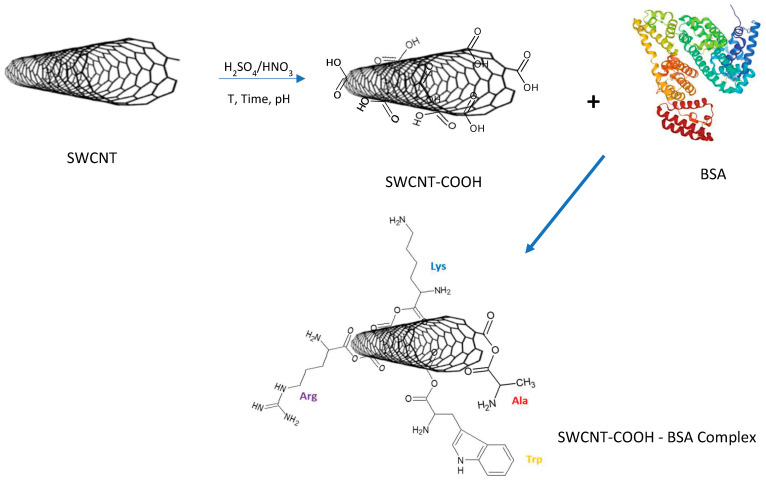
Non-covalent interaction mechanism in the formation of the SWCNT-COOH-BSA complex.

Furthermore, Dong et al. (2013) [125] by functionalizing SWCNTs and MWCNTs with HNO_3_/H_2_SO_4_, and evaluated their effect on biocompatibility on cells and enzymes. They concluded that controlled CNT oxidation allows removal of the metal catalyst, increases the number of functional groups on the CNTs with the ability to accept electrons, originates cleaved CNTs, and improves the solubility in aqueous environments. The biocompatibility with human epithelial cells and enzymes is also improved. Figure 9 illustrates the H-bond interactions in SWCNTs after acid functionalization and possible non-covalent interactions with the bovine serum albumin protein frequently utilized in drug delivery.

Salam and Burk (2017) [23] functionalized MWCNTs with HNO_3_ and their subsequent modification with ODA-octadecyl amine (CH_3_–(CH_2_)_17_–NH_2_) and PEG-polyethylene glycol, HO–(CH_2_–CH_2_–O)_n_–OH). The solubility of MWCNTs in different solvents was evaluated and they found that MWCNTs can be soluble in non-polar solvents such as dichloromethane due to the hydrophobicity of CNTs, but insoluble in water and methanol. However, this condition changed when functionalized with COOH, which caused the increase of MWCNTs hydrophilic character, resulting in them being soluble in water and methanol, but poorly soluble in hexane and dichloromethane. This same solubility was present in MWCNT-PEG. However, in MWCNT-ODA the hydrophilic character decreased so that the solubility in polar solvents decreased while it was increased in non-polar solvents. The functional groups carboxylate -COOH and amine -NH_2_ can be utilized with PEG without affecting the colloidal stability of the PEG nanoparticles in blood and plasma [126]. 

## 4. CNTs—Flavonoids

**Figure 10:**
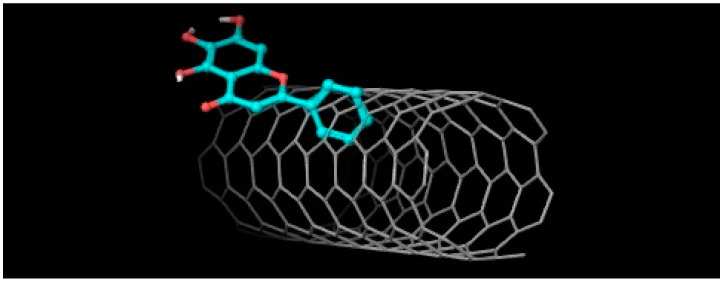
5,6,7-Trihydroxyflavone ― SWCNTs interaction. ΔG = −11.77 kcal/mol.

In studies in silico carried out by the author between 5,6,7-trihydroxyflavone (baicalein) and SWCNTs with the Autodock 4.0 program [127], a non-covalent interaction at ΔG = −11.77 kcal/mol was found (Figure 10). 

**Figure 11:**
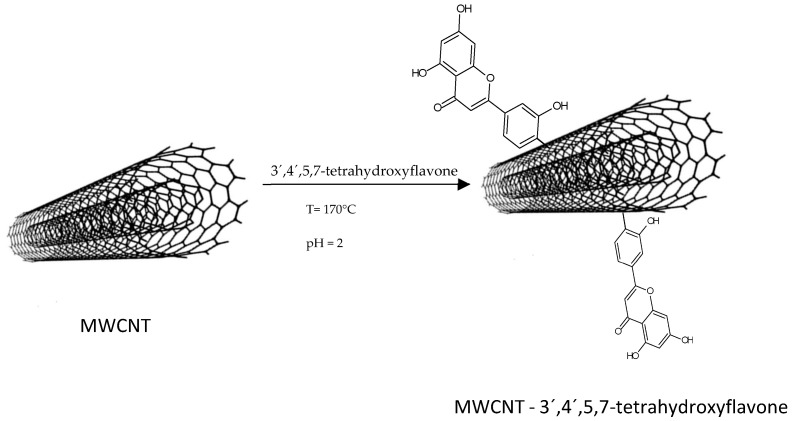
Reaction of 3′,4′,5,7-tetrahydroxyflavone—MWCNTs.

**Figure 12:**
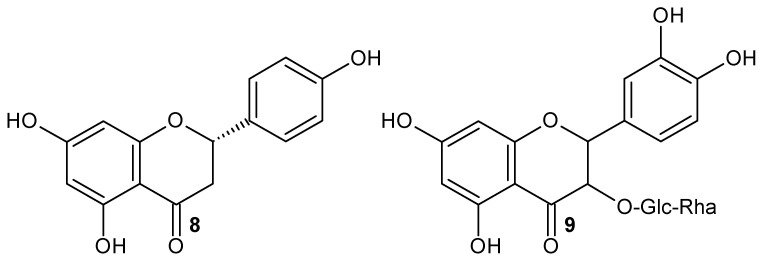
Naringenin **8** and rutin **9** structures.

The high capacity of CNTs to adsorb organic and inorganic molecules is mainly due to electrostatic interactions between the oxygen functional of the molecule and the adsorbent, but van der Waals forces and π-π stacking interactions between the aromatic rings of the molecule and the graphene mesh also contribute. Thus, the functionalization of CNTs with -OH, -COOH, and C=O groups is important [27]. However, in the following illustrative scheme (Figure 11), the interaction between 3′,4′,5,7-tetrahydroxyflavone and MWCNTs without functionalization is proposed:

Morais et al. (2020) [25] functionalized CNTs with the flavonoid naringenin-Ngn (**8**) (Figure 12) for targeted delivery into lung cancer cells. They found that in flavonoid functionalization, acid treatments modify the surface of CNTs, leading to the formation of oxygenated groups such as hydroxyl, ketones, carboxyl, and quinones on the surface of carbon nanotubes. This also originates the displacement of the bands corresponding to -OH and C=O at high frequencies, which indicates the formation of an intermolecular H-bond [128], and the presence of the band at 1470 cm^−1^ indicates the C=C elongation of the aromatic ring of the flavonoid [25]. They concluded that Ngn was adsorbed on the CNT surface by non-covalent interactions through H-bond, weak electrostatic interactions, and π-π stacking.

For their part, Gholizadeh et al. (2019) [29], evaluated the property of CNTs as adsorbents, a property related to their extensive surface area, electrostatic π-π interactions, and short equilibrium time. They utilized MWCNT-COOH as a method for the adsorption of flavonoids from orange peel. It was found that the presence of OH groups on the flavonoid molecule gives rise to H-bond interaction with the MWCNT-COOH surface. 

**Figure 13:**
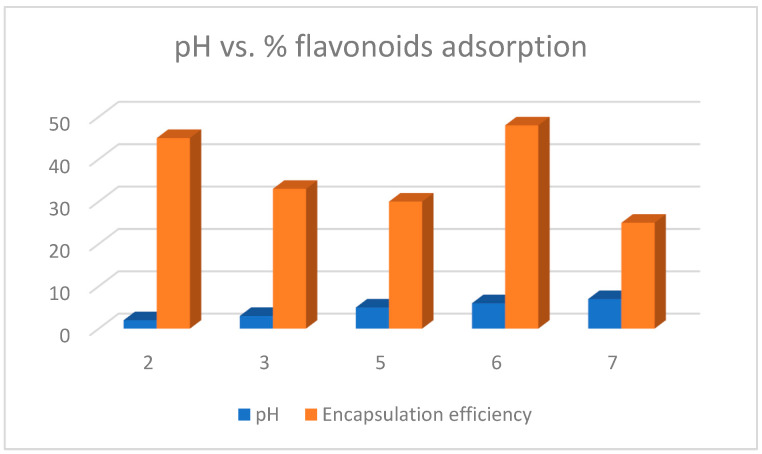
Effect of pH on flavonoid encapsulation efficiency in MWCNT-COOH.

It is to highlight that although Gholizadeh et al. (2019) [29] and Morais et al. (2020) [25] utilized MWCNT-COOH functionalized with the flavonoid naringenin (Table 7), the bands of the FT-IR spectra present different bands for the C=O and C=C groups. The peak corresponding to C=O is at 1639 cm^−1^ and that of C=C is at 1470 cm^−1^ for naringenin [25], whereas for the same flavonoid, these bands are at 1721 cm^−1^ and 1559 cm^−1^, respectively [29]. Flavonoids at high pH values dissociate into their anions where their functional groups are negatively charged or neutral; therefore, at high pH values the adsorption of flavonoids decreases due to the electrostatic repulsion of identical charges. Thus, Gholizadeth et al. (2019) [29] utilized MWCNT-COOH as an adsorption method for flavonoids such as rutin (**9**) (Figure 12) and naringenin. The adsorption percentage was determined using the following equation

$$\%A=\frac{A_{0}{-A}_{e}}{Aa}\times 100$$

where *A*_0_ and *A*_e_ are the initial and final adsorptions. They found that the percentage adsorption of flavonoids was pH-dependent (see Figure 13). So, at pH 2 the adsorption was 45% while at pH 7 it was 25%. However, Yang et al. (2019) [28] found that the adsorption of rutin on MWCNT-COOH was 48% at pH 6.0.

Concerning the effect of pH on flavonoid release from CNTs, Morais et al. (2020) [25] functionalized MWCNTs with the flavonoid naringenin and found the percentage release of flavonoid from the CNTs is pH dependent. Thus, at 9 h at pH 5, the release was 60.8%, while at pH 7.4 it was 83.5%. For their part, Gholizadeth et al. (2019) [29] also obtained a high 96.2% release of this same flavonoid from MWCNTs at pH 9. An 83% rutin release from MWCNT-COOH functionalized with polyamidoamine was obtained by Yang et al. (2019) [28].

Compounds such as polydopamine, cyclodextrin, chitosan, and polyacrylic acid have also been used to modify CNTs, as well as to change the functional groups and/or modify the adsorbent properties. The utilization of polyamidoamine dendrimers is frequent because of the amide units and the large number of amino groups in their structure, which make them suitable for bonding to other compounds and improving their adsorption capacity. Thus, the introduction of amino groups to an adsorbent could improve its adsorption capacity specifically with flavonoids.

Yang et al. (2019) [28] functionalized MWCNT-COOH with polyamidoamine dendrimers. They utilized N-hydroxysuccinimide (NHS) and N-ethyl-N-(3-(dimethylaminopropyl) carbodiimide (EDC) to improve the adsorption capacity and added FeSO_4_-7H_2_O and FeCl_3_-6H_2_O. These CNTs were utilized to extract the flavonoid rutin from a plant extract. FIT-IR spectra of MWCNT-COOH indicate a peak at 1631 cm^−1^ assigned to the carboxyl group on the surface of CNTs. Once functionalized, peaks at 1643 and 1558 cm^−1^ were found related to C=O at the N-H bond expressing primary and secondary amides. The peak at 2948 cm^−1^ corresponds to the C-H bond of the methylene groups and the O-H and N-H groups were present at peaks 3260 and 3425 cm^−1^, respectively. A signal peak at 579 cm^−1^ corresponds to the presence of Fe_3_O_4_. 

**Figure 14:**
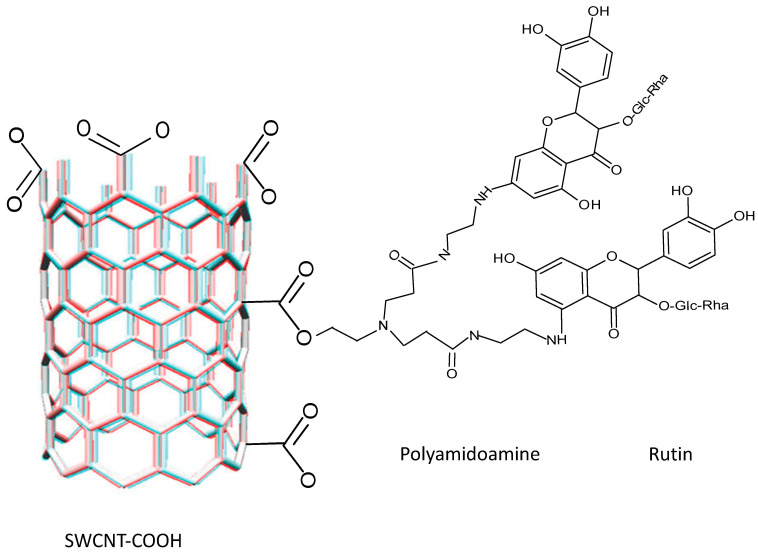
Non-covalent interaction mechanism between SWCNT-COOH―PAMAN―rutin.

The non-covalent interactions are based on van der Waals forces, π-π stacking, and are thermodynamically controlled [128]. The frequent presence of the six-membered ring of the flavonoid on the CNTs gives rise to a moderate π-π interaction with the aromatic rings of the rutin. In addition, polyamidoamine demonstrated that some amino and especially H-bond groups can form between the -NH2 of CNTs and the -C=O and -OH groups of rutin (see Figure 14). Therefore, π-π conjugation and H-bond interactions are important in the adsorption processes of flavonoid to CNTs functionalized with polyamidoamine; however, adsorption capabilities can be improved with NHS and EDC [28].

**Figure 15:**
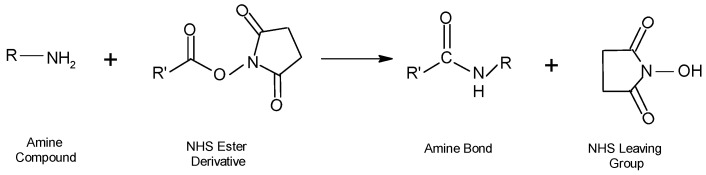
NHS ester-based reaction.

In the process of functionalization of NPs with amine groups by the addition of NHS, the carboxyl groups are activated and by reaction with the exposed amines of the proteins that are present on the surface, the molecule binds to the cell surface. For bioconjugated molecules, as well as for the conjugation of small molecules and synthetic macromolecules, this type of NHS ester-based reaction (reaction 1) is frequently utilized. (see Figure 15). 

An amide bond is usually formed with a protein or other molecule through aqueous two-phase coupling using EDC and NHS or sulfo-NHS. This involves the formation of a sulfo-NHS ester intermediate that has a higher reactivity than the EDC of the starting reagent for amine coupling. The utilization of NHS esters or reactive acyl groups of imidazole in organic solvent activation processes can be employed when there are stable particles in the solvent to obtain the same product with an amine-containing molecule [91]. 

**Figure 16:**
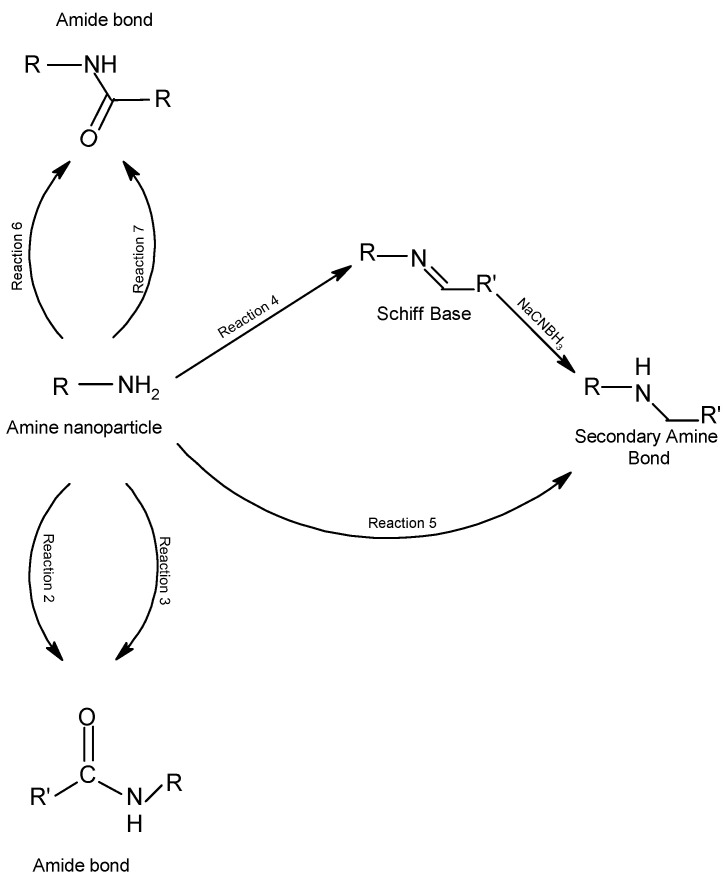
Covalent reactions through which amine-containing NPs may be coupled originating secondary amine bonds or amide bonds.

Amide or amine groups can be produced by several covalent reactions, as depicted in Figure 16. The acyl azides are activated carboxylates that react with primary amines to form amide bonds. Nucleophilic action on the electron-deficient carbonyl group occurs in the reaction between the acyl azide and an amino group (reaction 2). The higher the pH of the reaction medium, the higher the reactivity for both amine reactivity and hydrolysis, which leads to competition between acyl azide reactions and hydrolysis. An *N*-hydroxysuccinimide (NHS) ester is the main activating chemical needed to produce reactive acylating agents, since, for example, it forms an acylated product as in reaction 3, with compounds containing the ester-NHS. 

When sodium borohydride or sodium cyanoborohydride is added to a reaction containing an aldehyde and an amine as illustrated in Figure 16, a reduction of the Schiff base intermediate will occur, giving rise to a secondary amine bond (reaction 4) and a secondary amine, thioether, or ether bond formation occurs when an epoxy or oxirane group reacts with primary amines, sulfhydryls, or hydroxyl groups, respectively, in a reaction in which the opening of the β-ring gives rise to a hydroxyl group in the epoxy compound (reaction 5). In the reaction of succinic anhydride with a nucleophile, the anhydro ring is opened and an acylated product containing a carboxylate group is formed (reaction 6). In most cases the fluorophenyl ester is more stable in an aqueous solution against hydrolysis; however, upon reaction with amines at slightly alkaline pH conditions, amide-bonded NHS esters are produced (reaction 7).

In the case of histidine, the nitrogens of the imidazole ring side chain are acylated with the NHS ester, but they hydrolyze rapidly in aqueous media because the presence of imidazole in the reaction buffer increases the rate of hydrolysis. In contrast, stable bonds are produced in the reaction of these esters with primary and secondary amines. This occurs in the coupling of NHS-ester bonds with N-terminal α-amines and lysine chain amines in proteins [91].

Under alkaline conditions (pH 7.2–8.5), the ester-NHS-activated compound reacts with primary amines to form an amide bond and release NHS removed by dialysis or desalting. This reaction is also utilized at room temperature or at 4 °C for 0.5–4 h in a phosphate buffer at pH 7.2–8.0 to modify the primary amines on the cell surface.

Yang et al. (2019) [28] concluded that the ability of polyamidoamine-functionalized MWCNT-COOHs to adsorb flavonoids depends on the number of hydroxyl groups present on the flavonoid molecule.

Molecules that react with the -OH hydroxyl allow this group to be activated and facilitate its coupling to another functional group, forming a stable bond. Carbohydrates, polysaccharides, and glycoproteins can be coupled by reacting with hydroxyl groups. Similarly, organic molecules such as PEG, which contains hydroxyl, can also be conjugated into another compound. This allows these types of reactions to be generally employed in functionalization processes. 

Soddu et al. (2020) [129] evaluated the effect of the size and surface area of silica NPs and carbon NPs on the processes presented in human plasma. They evaluated the presence of functional groups on the surface of silica and carbon NPs on platelet-dependent and platelet-independent aggregation, platelet activation, and platelet adhesion. Both types of NPs had hydrophilic and negatively charged surfaces. Hydroxyl functional groups were present on the surface of silica NPs while carboxylic acid and phenolic groups were present on the surface of carbon NPs. Both NPs presented a zeta potential between −40 and 70 mV at pH 7.4. They observed that both NPs interacted with plasma proteins forming the corona protein. However, in carbon NPs this protein induces platelet-independent aggregation, but not in silica NPs. This difference is possibly due to the distribution of the protein molecules on the surface. 

For their part, Li et al. (2017) [95] functionalized MSNs with amino groups provided by 3-aminopropyltriethoxy silane, for the elaboration of baicalein NPs. They found that after the formation of MSNs, the surface of the NPs was changed from hydroxyl to amino groups, which caused a change in the zeta potential from negative −7.5 mV to positive +7.16 mV (in water). The hydroxyl groups present on the baicalein molecule can form H-bonds with the nitrogen of the amino groups. Such molecular interactions are the basis for the conduction and delivery of drugs from MSNs. Thus, the presence of functional groups on these nanoparticles can affect interactions with the cell.

**Figure 17:**
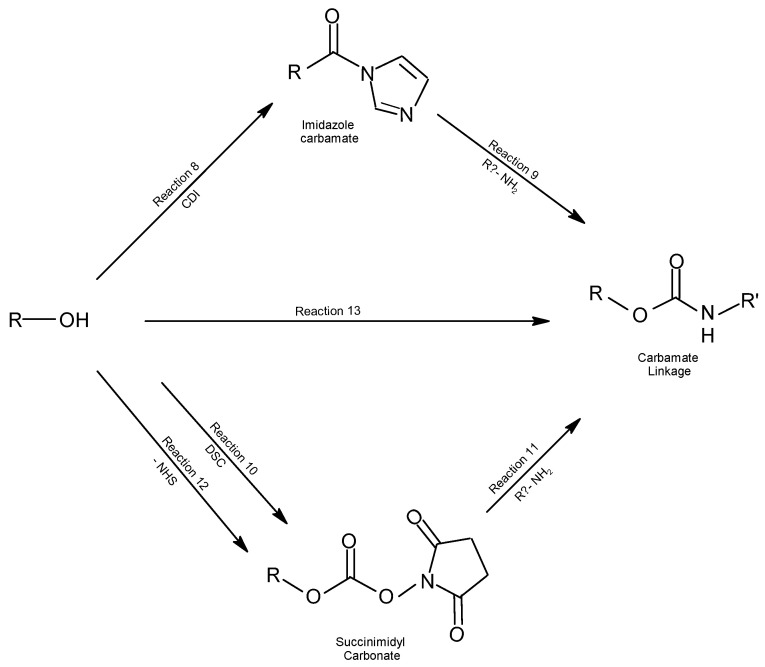
Covalent reactions through which hydroxyl NPs can be activated for ligand coupling.

Hydroxyl groups can be activated by several reaction mechanisms to form covalent bonds (see Figure 17). For interaction with ligands containing hydroxyl, amine, and thiol groups, activation processes with epoxy or vinyl sulfone are required, as well as cyanogen bromide, CDI, and DSC for coupling with amine molecules [91].

In the covalent interactions of hydroxyl groups, these may react with *N’,N*-carbonyl diimidazole (CDI), and the reactive intermediate imidazole carbamate is formed (reaction 8). Hydroxylated molecules react with amines to form stable urethane (*N*-alkyl carbamate) bonds, in which the amine action releases the imidazole but not the carbonyl (reaction 9). This type of interaction has been utilized both for the activation of chromatographic supports and for the activation of polyethylene glycol utilized for ligand immobilization and modification of amine molecules, respectively [92,93]. 

In nonaqueous environments, the *N’*,*N*-disuccinimidyl carbonate (DSC), containing two NHS esters, activates a hydroxyl group forming a succinimidyl carbonate derivative (reaction 10). However, in aqueous environments, DSC forms by hydrolysis of two *N*-hydroxysuccinimide (NHS) molecules with the release of CO_2_. The DSC-activated hydroxylated molecules can react with amine compounds, forming urethane-derived bonds or carbamate bonds (reaction 11) in stable cross-linked compounds. In non-aqueous environments, *N*-hydroxysuccinimidyl chloroformate can also activate the hydroxyl groups (reaction 12), and the reaction proceeds in the same manner as reaction 9. 

The formation of a urethane bond (carbamate) occurs by the rearrangement of acyl azides to form isocyanate and the reaction of this with a hydroxylated compound (reaction 13). 

Guzman-Mendoza et al. (2022) [123] functionalized MWCNT-COOH insulin from MWCNT purification with HNO_3_/H_2_SO_4_. They utilized insulin since the threonine residue present at the C-terminus of the protein binds through the hydroxyl groups of the lipase to the COOH groups on the MWCNT surface. The FT-IR results presented several peaks (Table 7), in addition peaks at 2875–2950 cm^−1^ correspond to C-H and a peak at 700 cm^−1^ corresponds to N-H of the protein residues.

## 5. Conclusions

Size variation, zeta potential, and PDI depend on the composition of the NPs. For the flavones 7,8-dihydroxyflavone (tropoflavin), 5,6,7-trihydroxyflavone (baicalein), 3′,4′,5,7-tetrahydroxyflavone (luteolin), and 4′,5,7-trihydroxyflavone (apigenin), the inclusion of poly(lactic-co-glycolic acid)] (PLGA) in both liposome and NP processing increases the size and entrapment efficiency. According to the analysis of flavone NPs, it was observed that when the composition of the NPs includes several compounds, a low potential is obtained, which indicates the formation of aggregates. The NP release percentage depends on the composition of the NPs and the pH of the medium, being more efficient in NPs functionalized with proteins and at high pH. A non-covalent H-bond, π-cation and π- π stacking-type molecular interactions, as well as covalent interactions with -OH, -NH_2_, C=C, and C-H functional groups are characteristic of flavone NPs. The functionalization of MWCNTs with acids alters the structure of MWCNTs, unlike MWCNTs functionalized with flavonoids and proteins. The percentage adsorption of flavonoids on MWCNT-COOH generally varies with pH; a higher percentage of flavonoid adsorption is obtained at low pH. The presence of an OH group on the surface of the NPs enhances properties for adsorption or bioconjugation processes with flavones. Some mechanisms of non-covalent and covalent interaction of NPs with amino -NH_2_ and hydroxyl -OH functional groups have been proposed.

## Figures and Tables

**Table 1:** Characteristics of some baicalein NPs.

NPs	Size (nm)	PDI	ζ (mV)	EE (%)	Reference
Liposomes	135–154	0.462–0.503	−1.89–−2.11	25.40–33.65	[96]
Mesoporous silica NPs (MSNs)	367 ± 94	---	−7.5 mV	---	[95]
Nanoemulsions (NE)	161.5 ± 5.5	0.107 ± 0.011	−22.4 ± 3.1 mV	98.2	[98]
Baicalein NPs	82.5 ± 1.7	0.12 ± 0.02	−1.5 ± 0.4 mV	86.2	[97]

**Table 2:** Effect of baicalein with nanocarriers on human cells.

NPs	Cells	Activity	Reference
Liposomes	Fibroblasts Hs68	Antioxidant	[96]
Mesoporous silica NPs (MSNs)	Primary human gingival epithelial cells (hGECs)	Anti-inflammatory	[95]
Nanoemulsion baicalein-paclitaxel co-encapsulation (PTX/BA NE)	Cancer cells MCF-7/Tax	Anti-tumor	[98]
Baicalein-paclitaxel dual ligand self-assembled NPs	Human lung cancer A549 cells	Anti-tumor	[97]

**Table 3:** Characteristics of some luteolin polymeric NPs.

NPs-Luteolin	Size (nm)	PDI	ζ (mV)	EE (%)	Reference
SLNs (solid lipid)	47 ± 0.51	0.247	−9.62	74.8	[103]
PLGA-NPs	184	---	27	91.8	[30]
Her-2-PLGA-NPs	203	---	19	90.4	[30]
PCL-NPs	161.8 ± 6.61	---	−31.2 ± 0.7	90.2	[105]
MPEG-PCL	38.6 ± 0.6	0.16	---	98.3	[106]
mPEG_5K_-PLGA_10K_	62.3 ± 0.01	0.098 ± 0.01	−6.12 ± 0.01	51.6	[107]
HRP/H_2_O_2_/PEG2050	234.8 ± 101.6	0.388	−36.2 ± 0.2	89.3	[108]
PLGA liposomes	99.1 ± 5.75	0.198	−12.5 ± 3.75	85.6	[109]
mPEG-PLGA-Eudragit S100	197.45 ± 20.09	0.22 ± 0.01	−23.5 ± 1.16	76.4	[104]

**Table 4:** Characteristics of flavone liposomes.

Flavone	Size (nm)	PDI	ζ (mV)	EE (%)	Reference
Tropoflavin	107.50–247.27	0.126–0.379	−19.57–−22.67	89.50	[91]
Baicalein	135–154	0.462–0.503	−1.89–−2.11	25.40–33.65	[96]
Luteolin	99.1 ± 5.75	0.198	−12.5 ± 3.75	85.6	[109]

**Table 5:** Some apigenin polymeric NP characteristics.

NPs	Size (nm)	PDI	ζ (mV)	EE (%)	Reference
Polymeric micelles	16.9	0.046	−5.87	96.36	[113]
API-GAL NPs	129	0.059 ± 0.007	−14	75.4	[114]
API-PLGA NPs	110	0.041 ± 0.004	−25	70.3	[114]

**Table 6:** Some characteristics of luteolin, apigenin, and hesperetin PLGA-NPs.

PLGA-NPs	Size (nm)	PDI	ζ (mV)	EE (%)	Reference
Luteolin	184	----	27	91.8	[30]
Apigenin	110	0.041 ± 0.004	−25	70.3	[114]
Hesperetin	260	0.355 ± 0.006	−42	80.5	[118]

**Table 7:** IR bands of functional groups on MWCNT-COOH.

	-OH	C=O	C=C	C-O	Reference
HNO_3_/H_2_SO_4_	3400 cm^−1^	1720 cm^−1^	1600 cm^−1^	1200 cm^−1^	[32]
Naringenin	3396 cm^−1^	1639 cm^−1^	1470 cm^−1^	---	[25]
Naringenin	3337 cm^−1^	1721 cm^−1^	1559 cm^−1^	1049 cm^−1^	[29]
Insulin	3300 cm^−1^	1733 cm^−1^	---	1239 cm^−1^	[123]
